# Super-Resolution Reconstruction Method of Pavement Crack Images Based on an Improved Generative Adversarial Network

**DOI:** 10.3390/s22239092

**Published:** 2022-11-23

**Authors:** Bo Yuan, Zhaoyun Sun, Lili Pei, Wei Li, Minghang Ding, Xueli Hao

**Affiliations:** School of Information Engineering, Chang’an University, Xi’an 710064, China

**Keywords:** image reconstruction, pavement crack image, super-resolution, deep Learning, GAN

## Abstract

A super-resolution reconstruction approach based on an improved generative adversarial network is presented to overcome the huge disparities in image quality due to variable equipment and illumination conditions in the image-collecting stage of intelligent pavement detection. The nonlinear network of the generator is first improved, and the Residual Dense Block (RDB) is created to serve as Batch Normalization (BN). The Attention Module is then formed by combining the RDB, Gated Recurrent Unit (GRU), and Conv Layer. Finally, a loss function based on the L1 norm is utilized to replace the original loss function. The experimental findings demonstrate that the self-built pavement crack dataset’s Peak Signal-to-Noise Ratio (PSNR) and Structural Similarity (SSIM) of the reconstructed images reach 29.21 dB and 0.854, respectively. The results improved compared to the Set5, Set14, and BSD100 datasets. Additionally, by employing Faster-RCNN and a Fully Convolutional Network (FCN), the effects of image reconstruction on detection and segmentation are confirmed. The findings indicate that the segmentation results’ F1 is enhanced by 0.012 to 0.737 and the detection results’ confidence is increased by 0.031 to 0.9102 when compared to state-of-the-art methods. It has a significant engineering application value and can successfully increase pavement crack-detecting accuracy.

## 1. Introduction

Many external variables, such as movement speed, equipment performance, relative light intensity, etc., interfere with pavement crack image collecting, resulting in poor image quality and drastically reduced detection and segmentation accuracy. Image super-resolution reconstruction [1] is proposed to improve the original image’s resolution and enhance the image’s content features, thereby resolving the faults produced by low-resolution images. This approach efficiently reduces hardware limits, enhances identification accuracy, and serves as a valuable reference for long-term pavement maintenance [2].

Previously, the network topology that resulted in good model performance was continually deeper. Despite the fact that images with a higher PSNR are recreated, texture details are not realistic or natural, and model degradation is a possibility. Excessive focus on the reconstructed image’s PSNR value while neglecting the visual effects’ performance results in extremely smooth texture detail.

Due to their remarkable generating capabilities, generative adversarial networks (GANs) theory and applications have rapidly expanded in recent years [3,4,5], and multiple variations [6,7,8,9] have been proposed and are frequently employed in diverse tasks of image processing. GANs are also employed in a number of areas [10,11,12,13,14] as important methods of virtual generation [15] and data set augmentation [16]. Christine et al. [17] proposed a DCGAN-based synthetic data method for improving the recognition of traffic signs. In this method, CNN models with different backbone networks and feature extractors are analyzed and discussed, and Resnet50 and Densenet are introduced into DNGAN to evaluate the generation effect. After mixing the synthetic image with the original image, the detection network is trained again, which improves the accuracy of traffic sign recognition performance and reduces the detection speed accordingly. Zhang et al. [18] proposed a permanent magnet motor fault diagnosis method based on DCGAN-RCCNN, which used DCGAN to generate virtual fault data, and used RCCNN to feature extract and classify stator current data, and finally realized the expansion of training samples. This method effectively solves the problems of insufficient fault data and difficult fault diagnosis of permanent magnet motors.

The Super-Resolution Using Generative Adversarial Networks (SRGAN) [19] was presented for image super-resolution reconstruction, and the results were impressive. SRGAN separates the network into generative and discriminant networks, allowing for increased image resolution and restoration of image detail characteristics without sacrificing PSNR.

In order to improve the recognition ability of low-resolution number plates, the problem of poor boundary and contrast is solved. Kabiraj et al. [20] used an enhanced super-resolution generative adversarial network to upscale from low resolution to high resolution to recover useful information that would improve the accuracy of number plate detection and recognition. They used the residual module to improve the native dense blocks of the generative adversarial network and combined the OCR model to segment the number plates to improve the detection performance of number plate information. Rashid et al. [21] applied this method to enhance the spatial resolution of two-dimensional MR images. By modifying ESRGAN’s network structure, it can train 2D Magnetic Resonance images (MRI). Xu et al. [22] proposed a new network architecture based on the principle of SRGAN: Enhanced High-Resolution Semantic Network (EHRS-Net). This method maintains and enhances the details of the feature map through the convolution process to improve the overall crack detection accuracy. By evaluating two pavement crack datasets, it is proved that the proposed pavement crack detection method is superior. Pei et al. [11] expanded the crack training set based on DCGAN, used Faster-RCNN to train the data set before and after the expansion, and found that the detection accuracy improved.

Even though SRGAN can generate sharper images compared to other networks, its network structure is complex. If the depth of the model is raised blindly for special tasks, the network parameters will drastically increase, and the performance improvement will be restricted. The analysis found that SRGAN has the following problems: (1) SRGAN is mainly composed of residual blocks of the same structure, consisting of two convolutional layers and two BN layers. As discussed in [23], the computational cost soars when the number of network layers increases. (2) SRGAN inherits the characteristics of GAN and pays more attention to the authenticity of the generated images while ignoring the differences in the details of the generated images. It leads to the problem that visual perception and practical application cannot be better satisfied. (3) The performance of pavement crack detection is highly dependent on the image quality sent into the network. The direct use of the original SRGAN, although the image’s resolution has been improved, there is still room for improvement in the reconstruction of crack characteristics.

This research is presented to solve the above problems and combine the engineering needs of pavement detection. The creativity of the research mainly lies in the super-resolution model based on the GAN and the attention mechanism. The main technical contributions are as follows:

- Improved the non-linear network in the generator to create an attention recurrent network that removes the Batch Normalization (BN) and is fused by the Residual Dense Block (RDB), the Gated Recurrent Unit (GRU), and Attention. The improved generator pays more attention to crack features, and the reconstructed crack images are more detailed. This research aims to solve the shortcomings of high PSNR value but imperfect detail reconstruction in the original network reconstruction process.
- Replace the original GAN’s loss function with an L1 norm-based loss function. Using this loss function enhances the generator’s learning ability while also enhancing the image reconstruction quality.
- For the unique field of pavement crack image super-resolution applications, not only the PSNR and SSIM metrics are adopted, but also Faster-RCNN (the detection model) and FCN (segmentation model) are utilized to evaluate the detection and segmentation effects of reconstructed high-resolution images. These detailed evaluation methods can more comprehensively verify the methods’ effectiveness and provide reference ideas for other research in this field.

## 2. Methodology

This research incorporates the attention mechanism into the SRGAN model to address the model’s weaknesses. In terms of parameter control, a recursive structure is utilized to share network parameters so that the parameter scale does not expand dramatically as the network depth grows. In the performance of image details, feature extraction is performed on low-resolution images, and the attention mechanism is employed to distinguish between low-frequency information and high-frequency information.

### 2.1. Attention Generative Adversarial Network Structure

**Figure 1:**
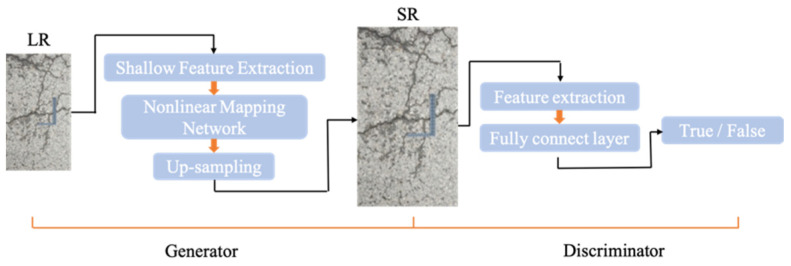
The structure of the attention generative adversarial network.

A generator and a discriminator are often used in GAN. The attention mechanism and recurrent network are introduced into the original network, and the components of GAN are redesigned. The upgraded component allows the super-resolution reconstruction model to combine the attention mechanism’s visual performance advantage with the recurrent network-sharing parameters’ feature. Figure 1 depicts the design model’s general structure.

The generator’s major components are shallow feature extraction, nonlinear mapping network, and up-sampling. The discriminator receives the super-resolution reconstructed image as input, which consists mostly of feature extraction and complete connection. It is used to determine whether the generator’s output and the real label are similar and whether the result is True or False.

### 2.2. Generator Design

**Figure 2:**
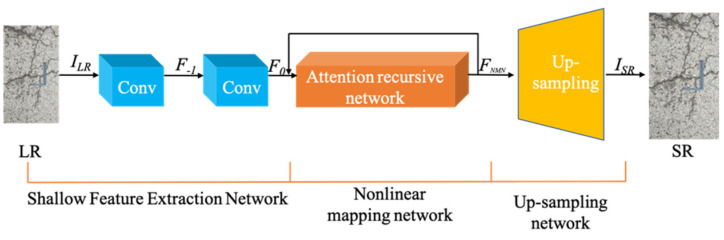
Generator structure with attention recursive network.

The shallow feature extraction network, nonlinear mapping network, and up-sampling network are the primary components of the generator. As illustrated in Figure 2, the nonlinear mapping network is an attention recurrent network.

$I_{LR}$ represents the low-resolution image, and $I_{SR}$ represents the result after network reconstruction. The low-resolution image input by the network extracts shallow feature information through the Shallow Feature Extraction Network, mainly composed of two convolutional layers (Conv). The first Conv layer extracts the feature $F_{-1}$ from the $I_{LR}$, and the second performs further shallow feature extraction on $F_{-1}$, and the output is $F_{0}$, as shown in Equations (1) and (2), respectively.

(1) $$F_{-1}=H_{SFE1}\left(I_{LR}\right)$$

(2) $$F_{0}=H_{SFE1}\left(F_{-1}\right)$$

**Figure 3:**
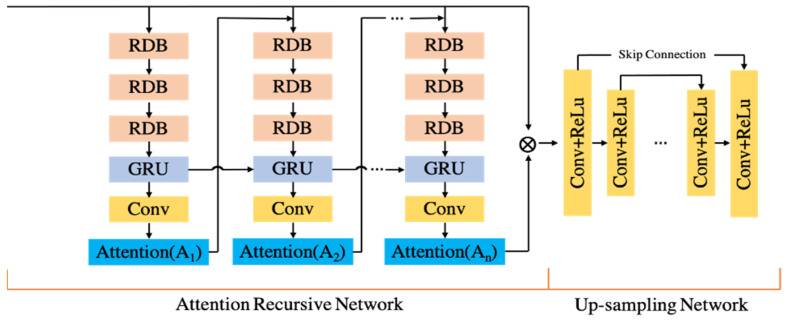
Attention recursive network structure design.

$H_{SFE1}(\cdot )$ and $H_{SFE2}(\cdot )$ serve as the convolution operations of the shallow feature extraction network. After the original input LR is subjected to shallow feature extraction, its output $F_{0}$ is sent to the attention recurrent network as a new input. The structure of the attention recurrent network is shown in Figure 3. The main purpose of the attention recurrent network is to extract the texture details that need to be recovered from the input image. The extraction results are enhanced by subsequent nonlinear mapping and used to generate high-resolution images. Therefore, the effect of reconstructing the image depends on the quality of texture detail extraction by the attention recurrent network.

(1) RDB: To replace the original structure, the redesigned dense residual block is shown in Figure 3. The BN layer creates pseudo-textures in the output images when the statistics of the training and test datasets change significantly, reducing the generalization ability. The BN layer is deleted to stabilize the network’s training, minimize computational complexity, and reduce computational overhead. RDB achieves feature fusion and dimensionality reduction by merging residual blocks with skip connections [23], resulting in dense blocks. To boost network capacity, the dense residual block not only preserves the feedforward information, but also fully extracts the local feature layer information.

(2) GRU: In Attention Recurrent Networks, GRU are introduced. As illustrated in Equations (3)–(6), the most significant structures are gate structures, namely *Reset Gate r*_t_ and *Update Gate z_t_*.

(3) $$r_{t}=\sigma \left(W_{r}\left[h_{t-1},x_{t}\right]\right)$$

(4) $$z_{t}=\sigma \left(W_{z}\left[h_{t-1},x_{t}\right]\right)$$

(5) $${\hat{h}}_{t}=\tan h\left(W\left[r_{t}\;*\;h_{t-1}\right],x_{t}\right)$$

(6) $$h_{t}=\left(1-z_{t}\right)\;*\;h_{t-1}+z_{t}\;*\;{\hat{h}}_{t}$$

In the hidden layer, $h_{t-1}$ is the state at the previous time, $h_{t}$ is the state at the current time, and ${\hat{h}}_{t}$ is the updated state at the current time. $W_{z}$ produces the weight corresponding to the update state, $W_{r}$ represents the weight in the reset state, and * represents the convolution operation. To produce 2D attention images, the GRU’s output features are fed to successive convolutional layers. The resulting attention image is concatenated with the input image at each time step during training and utilized as the input to the next layer of the recurrent network. As shown in Equation (7), it is assumed that the output of the recurrent attention network after n layers is $F_{n}$.

(7) $$F_{n}=H_{ATT,n}\left(F_{n-1}\right)=H_{ATT,n}\left(H_{ATT,n-1}\left(\cdots \left(H_{ATT,1}\left(F_{0}\right)\right)\cdots \right)\right)$$

$H_{ATT}$ represents the recurrent attention network function, and n represents the number of layers of recursion. Feature mapping is performed after the texture detail features are extracted through the attention recurrent network. The designed feature mapping network has 8 Conv + ReLUs structures, and skip connections are added to improve the stability of network training, as shown in the right half of Figure 3. The feature map network is shown in Equation (8):

(8) $$F_{NMN}=H_{NMN}\left(F_{n}\right)$$

*F_NMN_* is the output result of using the nonlinear network function $H_{NMN}$. The up-sampling network consists of convolutional layers before generating the high-resolution image to obtain the super-resolution image output scaled by a factor of 4 in Equation (9):

(9) $$I_{SR}=H_{UPS}\left(F_{NMN}\right)$$

$H_{UPS}$ is the up-sampling function, and $I_{SR}$ is the super-resolution image output.

**Figure 4:**
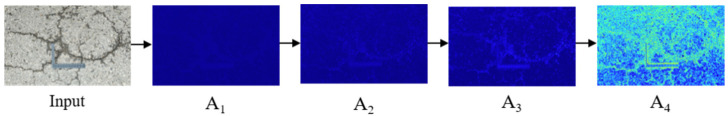
Attention map at different stages.

(3) Attention: The attention feature of each layer of a recurrent network is a matrix with values between 0 and 1. The stronger the associated attention, the higher the element values in each matrix. The first four Attention (*A*_1_–*A*_4_) in the Attention recursive network are selected for the output of the attention feature map, and they are colored using pseudocolor, as shown in Figure 4, where $\;A_{n}$ are the generated visual attention maps. As recursion increases, attention feature maps highlight texture details and edges.

The generated visual attention map output by the first attention module *A*_1_ is basically blue. When recursive to Attention (*A*_4_), *A*_4_ corresponding to the input (the crack target area) is yellow, while the background is still blue. Therefore, the feature maps output by the four attention modules is weak in blue and strong in yellow.

### 2.3. Discriminator Design

**Figure 5:**
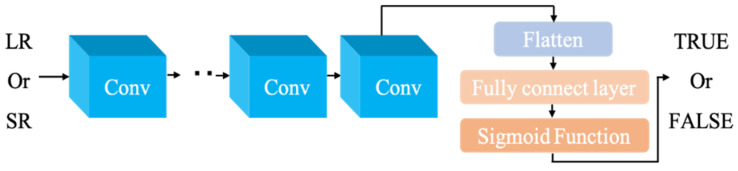
Discriminator structure.

After redesigning the generator, the discriminator needs to be improved to match the new generator in Figure 5. GAN uses a discriminator to judge the real and generated images, which are then fed back to the generator. The visual characteristics of the high-resolution-produced image to be assessed are extracted using seven layers of convolutional layers, then dimensionally flattened. Lastly, the results are discriminated using the fully connected layer (FC) and the Sigmoid function.

In the first training, the performance of the original generator was considerably inferior to that of the discriminator, causing the model to crash. To avoid this from happening, the loss function must be enhanced. The original loss function is replaced with a new loss function based on the L1 norm, which includes the discriminator’s reconstruction error. The loss function improves the learning ability of the generator while improving the image quality.

The image pixel-level error is considered to obey the Gaussian distribution, and the loss function based on the L1 norm is defined in Equation (10).

(10) $$L\left(I\right)=\left|I_{HR}-G\left(I_{LR}\right)\right|$$

$I_{HR}$ indicates the high-resolution images, and $I_{LR}$ indicates for low-resolution images. The optimization rules of the generator and discriminator loss functions that need to be iterated are expressed in Equations (11)–(15):

(11) $$\left\{\begin{array}{c} L_{D}=L_{D_{r}}-k_{t}L_{D_{f}},for\theta_{D}\\ L_{G}=L\left(G\left(z\right)-x\right),for\theta_{G}\\ k_{t+1}=k_{t}+\lambda_{k}\left(\gamma L_{D_{r}}-L_{G}\right) \end{array}\right.$$

(12) $$L_{D_{f}}=L\left(D\left(x;\theta_{D}\right)-x\right)$$

(13) $$L_{D_{f}}=L\left(D\left(y;\theta_{D}\right)-y\right)=L\left(\left(D\left(G\left(z;\theta_{G}\right)\right)-G\left(z;\theta_{G}\right)\right);\theta_{D}\right)$$

(14) $$y=G\left(x;\theta_{D}\right)$$

(15) $$\gamma =\frac{E\left[L\left(G\left(z\right)\right)\right]}{E\left[L\left(x\right)\right]}$$

*x* performs a high-resolution image, z represents a low-resolution image, y is a super-resolution image, $L_{D_{f}}$ represents the loss of a high-resolution image by the discriminator, and $L_{D_{f}}$ indicates a loss of a low-resolution image. $\lambda_{k}$ represents the increment of k, $k_{t}$ means the result of the t-th iteration of k, and the change of the value of k can be used to improve the learning ability of the generator. The γ is the ratio of the expected value of the super-resolution image error to the expected value of the high-resolution image. The value of this parameter can improve the quality of the generated image.

## 3. Experimental Evaluation and Results Analysis

### 3.1. Training Parameter Setting

**Figure 6:**
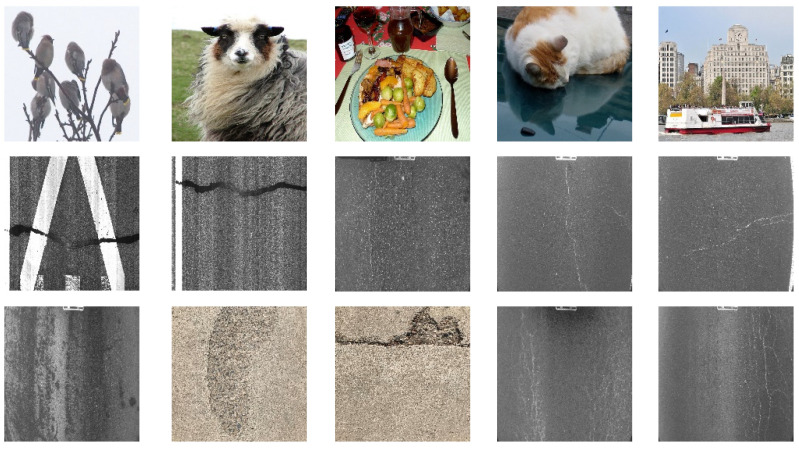
Training dataset.

The dataset, the number of network layers, and the convolution kernel size are all elements that affect network performance. In this experiment, the training set includes 800 images from the ImageNet public dataset and 300 images from self-built pavement crack datasets in Figure 6. The self-built dataset mainly contains three types of pavement cracks: horizontal, longitudinal, and reticular. A Garmin VIRB professional sports area scan camera is used for sampling, and a 100W LED supplementary light source is used to avoid the interference of road shadows. The calibration shooting area is 1.5 m wide and 1 m long, and the resolution is 2048 × 1024. The camera is placed 1 m from the ground to shoot vertically. To avoid shooting shadows caused by direct sunlight on sunny days, try to choose cloudy days and set the exposure time to 300 us. The training set’s images are downsampled at a predetermined ratio of four times (4).

SRCNN [24] with different numbers of convolution kernels and varied convolution sizes and DRCN [25] with varying convolution sizes are evaluated independently using the PSNR measure to assess the training conditions.

Set the number of convolution kernels in the first layer to $n_{1}$ and the second layer to $n_{2}$, and conduct comparative experiments in three cases: (i)$\;n_{1}$ = 32, $n_{2}$ = 16; (ii)$\;n_{1}$ = 64, $n_{2}$ = 32, (iii) $n_{1}$= 128, $n_{2}$ = 64. The size of the convolution kernel’s first layer of the convolution kernel is $f_{1}$= 9, and the last layer is $f_{2}$ = 5. The results are shown in Table 1. When the first layer and the second layer have 64 and 32 convolution kernels, respectively, the PSNR value is the highest. The network produces high-resolution images with the greatest possible performance and optimal performance.

Based on the above set, expand $f_{1}\;$= 11, $f_{2}$ = 7, and compare other parameters unchanged. The result is 25.91 dB, which is only 0.08 dB higher than the previous result. However, after expanding the convolution kernel’s size, the network’s training parameters increase greatly.

Set the size of the recursive layer convolution kernels to 3×3, 5×5, and 9×9 for DRCN. Table 2 shows that when the kernel size is 3×3, the network can get the best performance of 27.29 dB.

SRGAN’s generator is composed of five residual blocks with the same structure. In each residual block, the size of the kernel in the convolution layer is set to 3×3, 5×5, and 9×9, respectively. Table 3 shows that when the kernel is 3×3, the PSNR value is greater, resulting in 29.24 dB.

Based on the pre-training experimental findings mentioned above, the improved method sets all convolutional layers in the attention feature extraction module of the generator to 64, the size of the convolution kernel is 3×3, and the up-sampling network utilizes a 3×3 size convolution kernel. The number of attention feature extraction modules are set to 4 (4× magnification). The Adam optimizer’s learning rate for network training is set to 1$\times 10^{-4}$.

### 3.2. Experimental Evaluation

#### 3.2.1. Evaluation Index

The evaluation index is to achieve an evaluation result consistent with human visual perception as much as possible. The major method is a full-reference evaluation based on the direct difference between all pixels in the image. Therefore, the first step is to get the data for all the pixels, and the second step is to diff all the pixels, find the differences between the images, and sort them to evaluate the quality of the images. Mean Square Error (MSE), PSNR, and SSIM are the most frequently utilized full-reference assessment techniques. PSNR and SSIM are frequently used as assessment indicators in image super-resolution reconstruction.

(1) PSNR.

The PSNR is the ratio of a signal’s maximum possible power to the destructive noise power that impacts its accuracy. Equation (16) defines PSNR as follows:

(16) $$PSNR=10ln\frac{L^{2}}{MSE}$$

The meaning of L refers to the maximum grayscale an image can represent, and different images have different L values. If an eight-bit binary system represents the pixel information of an image, then L=255.

(2) SSIM.

SSIM is different from the direct solution method of MSE and PSNR. SSIM introduces the specific structure in the image into the evaluation method, strips off the influence factors of different illumination and contrast between images, and evaluates the difference in image quality through specific structural differences between images. SSIM is defined in Equation (17):

(17) $$SSIM=\frac{\left(2\mu_{x}\mu_{y}+C_{1}\right)\left(2\sigma_{xy}+C_{2}\right)}{\left(\mu^{2}{}_{x}+\mu^{2}{}_{y}+C_{1}\right)\left(\sigma^{2}{}_{x}+\sigma^{2}{}_{y}+C_{2}\right)}$$

x and y represent the image to be evaluated and the reference image, respectively. μ and σ, respectively, refer to the mean and variance between image pixels. $C_{1}$ and $C_{2}$ are constants, and they are set to $C_{1}={\left(k_{1}L\right)}^{2}$, $C_{2}={\left(k_{2}L\right)}^{2}$, where $k_{1}=0.01$,$k_{2}=0.03$, L=255. The maximum value of SSIM is 1, which signifies that the image to be assessed is precisely the same as the original image. When the estimated value of SSIM approaches 1, it indicates that the similarity between the images is rising, while when it approaches 0, it indicates that the images are becoming increasingly dissimilar.

#### 3.2.2. Performance Comparison and Analysis

**Figure 7:**
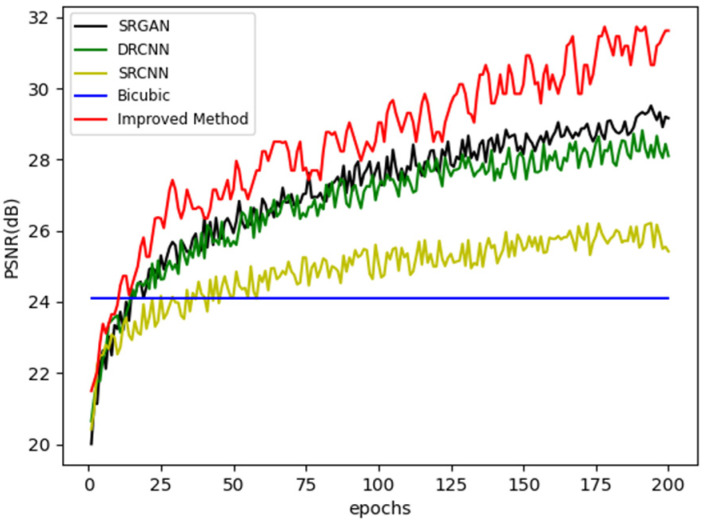
Comparison of different super-resolution methods.

Bicubic [26], SRCNN [24], DRCN [25], SRGAN [27], and the improved method are compared. Firstly, the convergence speed and final accuracy of different networks are compared to demonstrate the performance advantages of the innovative model. The PSNR values of various epoch outcomes during the training process of various approaches are compared in Figure 7. The improved approach provides the quickest convergence time and the highest final PSNR value. It also demonstrates that the new approach has a better level of training stability.

**Figure 8:**
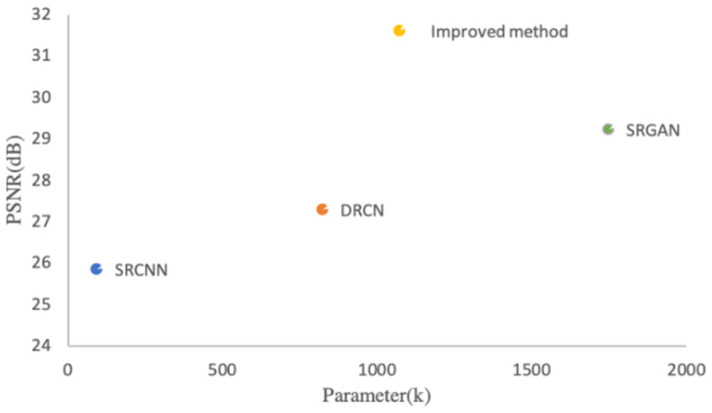
Comparison of network parameters and PSNR.

Secondly, the parameter complexities of various networks are compared. Figure 8 provides a comparison of network parameters and method performance. Under the same test set, the improved method’s parameters are about $1.1\times 10^{6}$. Compared with the SRGAN method, the parameters of the improved method are reduced by about $0.65\times 10^{6}$. However, the PSNR value of the image reconstructed by the improved method is 2.01 dB higher. Compared with the SRCNN and DRCN methods, although the improved method has more network parameters, the number of network layers has increased significantly, which also improves the final PSNR value compared to the shallow network.

Then, the experiment adopts a fixed four-times image magnification, and tests are carried out on Set5, Set14, BSDS100 and self-built pavement crack test data, respectively. The final performance results of different methods are shown in Table 4. On the self-built fracture data, PSNR and SSIM reach 29.21 and 0.854, respectively. In addition, the improved method has the best performance under different datasets.

**Figure 9:**
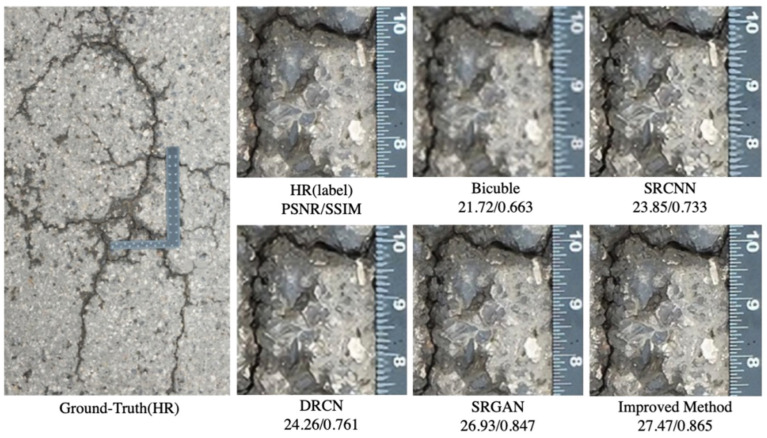
Reconstruction results comparison.

Real-world photos of pavement cracks are used to evaluate the improved approach. The enhanced method’s PSNR value is 27.47 dB, and the SSIM value is 0.865, which are 0.54 and 0.018 higher than SRGAN, respectively, as shown in Figure 9. The enhanced approach restores the texture features of the pavement better, and the pavement cracks are also sharper and clearer.

### 3.3. Evaluation Based on Faster-RCNN and FCN

The novel method’s main goal is to provide technical support for pavement crack identification. Therefore, the detection and segmentation effect of pavement image super-resolution reconstruction is the key to evaluating the improved method, which directly proves its reliability and stability in the application field.

The F1 index is utilized as a comprehensive evaluation indicator to evaluate the accuracy of the crack detection and segmentation results in this case. To calculate the F1 value, precision and recall must be obtained ahead of time. In Equations (18)–(20), they must employ four types of pixel data for segmentation results: true positive (TP), true negative (TN), false positive (FP), and false negative (FN).

(18) $$precision=\frac{TP}{TP+FP}$$

(19) $$recall=\frac{Tp}{Tp+FN}$$

(20) $$F1=2\times \frac{precision\;*\;recall}{precision+recall}$$

#### 3.3.1. Pavement Crack Detection Evaluation

**Figure 10:**
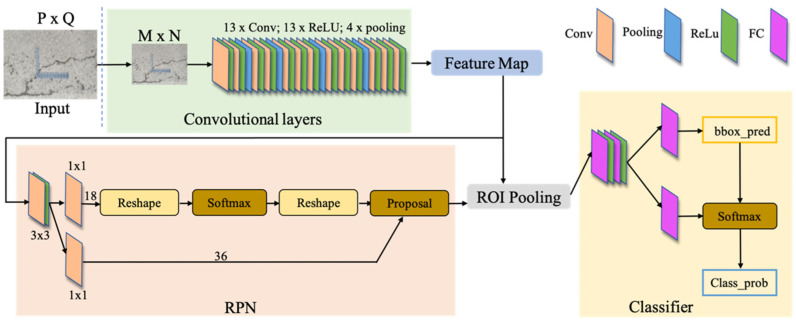
Pavement crack detection network structure.

The basic structure of the pavement crack detection method depends on the Faster R-CNN widely used in the field of image detection [28]. The model is shown in Figure 10, and is mainly composed of four parts: Conv layers, Region Proposal Networks (RPN), Region of Interest (RoI) Pooling, and Classifier.

This network inputs the image into the Conv layers. The size of the input image is uniformly adjusted to M×N. Through the VGG16-based network for feature extraction. The network comprises 13 convolutional layers, 13 ReLU layers and four pooling layers. After feature extraction, the feature map is output. Feature maps will be input into the RPN network. After convolution by a 3×3 convolution kernel, it is divided into two branches. The upper branch classifies the candidate frame through Softmax to obtain the detection target and background. The lower branch is used to calculate the offset of the candidate frame to adjust the accurate position of the candidate frame (Proposal). Immediately after that, the Feature map and proposal output from the previous step will be input into RoI Pooling simultaneously. The main function of RoI Pooling is to adjust to a unified feature map size. Finally, the Classifier judges the feature map output by RoI Pooling and adjusts the position and size of the detection frame again to achieve a more accurate result.

Table 5 shows the precision, recall and F1 of crack images of different super-resolution methods when Confidence_threshold is 0.5 and 0.75.

The results show that when Confidence_threshold = 0.5, although the recall of the dataset after the improved method is similar to the rest of the methods, the precision reaches 91.02%, and the F1 value is as high as 0.9027. The super-resolution images obtained by the improved method maintain a high recall rate when passing through the detection network, the model detection accuracy is higher, and the comprehensive performance is better. To further improve the detection requirements, the Confidence_threshold value is increased to 0.75. Even though the precision and recall of the super-resolution image of the method in this paper are reduced by 10.29% and 16.8%, respectively, compared with the Confidence_threshold = 0.5. However, the F1 reaching 0.7868 is higher than the rest of the methods. It is verified that the improved method has more stable properties and engineering application value under higher requirements.

Three images were randomly sampled containing horizontal, longitudinal, and reticular cracks. They are processed by different super-resolution methods and then fed into the Faster-RCNN for crack detection. The detection effect and confidence are shown in Table 6.

#### 3.3.2. Pavement Crack Segmentation Evaluation

**Figure 11:**
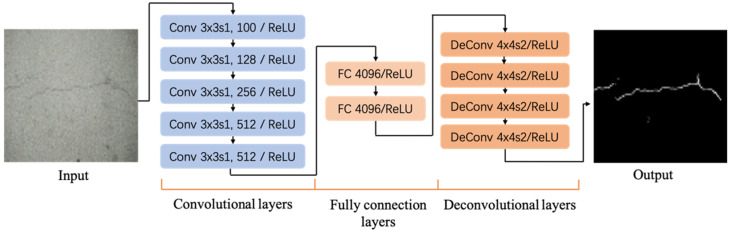
Pavement crack segmentation network structure.

To assess the reconstruction effect of super-resolution crack images, the most representative FCN was chosen, which has been widely utilized in image segmentation. This end-to-end network can automatically segment the cracks from the input pavement crack image, eliminating useless information and noise interference. The FCN must first adjust the size of the input image, use the convolutional layer to extract features from the image to generate a feature map, use the FC to identify pavement cracks, and finally restore the cracks to the original input image after the deconvolution layer size. The network is mainly composed of five convolutional layers, two fully connected layers and four deconvolutional layers. The specific structure is shown in Figure 11. The pavement crack image after super-resolution reconstruction is fed into the FCN network, and the effectiveness of the designed super-resolution model is evaluated by comparing crack segmentation accuracy.

For super-resolution, the self-built pavement image data is reconstructed using several methods, and the results are sent into the FCN network for segmentation. Table 7 shows the relevant F1 and SSIM indications. The improved method’s F1 value is 0.737, up 0.012 from SRGAN. The SSMI value increased by 0.0043 to 0.9945, compared to SRGAN’s 0.0.9902.

**Figure 12:**
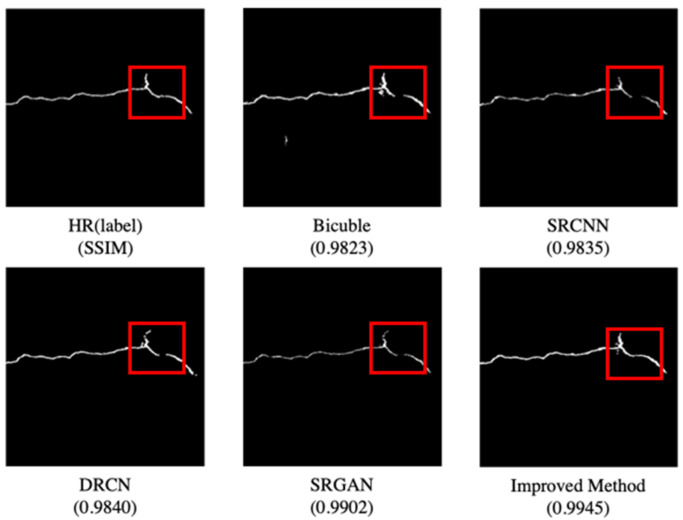
Pavement cracks segmentation comparison.

The crack segmentation effect is seen in Figure 12. After going through the FCN segmentation network, the enhanced method’s reconstructed image has a more complete and continuous result (*red area*). The SSIM value of the segmented image and label is 0.9945, implying that the enhanced method’s crack image is the most similar to the original label in the segmentation result.

## 4. Conclusions and Future Work

This research proposes a GAN-based super-resolution reconstruction method for pavement cracks. The reconstructed crack image by the improved method is compared with other methods through an objective evaluation index, crack detection and segmentation network. The main conclusions are as follows:

(1) The research adopts RDB, GRU and Attention to upgrade the nonlinear network of the original generator and remove the BN layer. The feature learning ability of the network for the crack foreground is improved, and the network learning parameters aer reduced further. In the training process of the network, the loss function based on the L1 norm is introduced to speed up the convergence of the network and make the network pay more attention to the loss of detailed features of cracks, avoiding the defect of only focusing on the improvement of PSNR and SSIM indicators but insufficient detail reconstruction.

(2) Comparing with Bicubic, the SRCNN, DRCN and SRGAN methods on Set5, Set14, BSD100 and self-built crack datasets. Compared with the previous best results, the improved method improves the PSNR of the above dataset by 2.38, 1.91, 1.38 and 0.68, respectively. SSIM increased by 0.062, 0.043, 0.036 and 0.027, respectively. The reconstructed PSNR and SSIM of the self-built fracture dataset are 29.21 and 0.854, respectively.

(3) For the actual engineering scene, the image reconstructed by the self-built crack dataset with the improved method is fed into Faster-RCNN and FCN for detection and segmentation. The comprehensive detection accuracy is 91.02%, and the F1 reaches 0.9027. The F1 and SSIM of the segmentation effect are 0.737 and 0.9945, respectively.

Currently, the training model requires that the image magnification be chosen in advance, which is inflexible in practice. As a result, future research will focus on developing a super-resolution reconstruction model applicable to any magnification. At the same time, pavement noise (shadows, uneven illumination, etc.) can also indirectly impact pavement crack detection and segmentation performance. Preserving pavement crack features and eliminating noise interference as much as possible during the super-resolution processing of images will be the focus of our next research work.

## Figures and Tables

**Table 1:** Comparison of PSNR values of SRCNN reconstruction. Results with different numbers of convolution kernels.

*n*_1_	*n*_2_	PSNR (dB)
32	16	25.17
64	32	**25.83**
128	64	25.20

**Table 2:** PSNR values of DRCN with different kernels.

Convolution Kernel Size	PSNR (dB)
3 × 3	**27.29**
5 × 5	26.86
9 × 9	27.05

**Table 3:** Comparison of PSNR values of SRGAN reconstruction results with different convolution kernel sizes.

Convolution Kernel Size	PSNR (dB)
3 × 3	**29.24**
5 × 5	28.59
9 × 9	28.94

**Table 4:** PSNR and SSIM comparison on different data sets.

Method	Evaluation Index	Dataset			
		Set5	Set14	BSD100	Self-Built Crack Dataset
Bicubic [26]	PSNR (dB)	24.72	22.56	24.99	21.68
	SSIM	0.695	0.631	0.704	0.605
SRCNN [24]	PSNR (dB)	25.83	23.16	25.49	22.63
	SSIM	0.811	0.726	0.785	0.687
DRCN [25]	PSNR (dB)	27.29	27.02	26.66	26.27
	SSIM	0.832	0.810	0.794	0.759
SRGAN [27]	PSNR (dB)	29.24	27.46	28.81	28.53
	SSIM	0.841	0.822	0.836	0.827
Improved method	PSNR (dB)	**31.62**	**29.37**	**30.19**	**29.21**
	SSIM	**0.903**	**0.865**	**0.872**	**0.854**

**Table 5:** Self-built dataset pavement crack detection matrix.

Method	Confidence_Threshold	Precision (%)	F1	Recall (%)
Bicubic	0.5	76.74	0.7682	76.91
SRCNN	0.5	82.97	0.8582	88.84
DRCN	0.5	84.68	0.8643	88.26
SRGAN	0.5	87.92	0.8838	88.84
**Improved method**	**0.5**	**91.02**	**0.9027**	**89.53**
Bicubic	0.75	63.77	0.6531	66.92
SRCNN	0.75	74.03	0.7668	79.53
DRCN	0.75	77.75	0.6890	61.87
SRGAN	0.75	79.20	0.7699	74.90
**Improved method**	**0.75**	**80.73**	**0.7868**	**76.73**

**Table 6:** Comparison of crack detection results and confidence.

Method	Horizontal	Longitudinal	Reticular
Benchmark (HR)	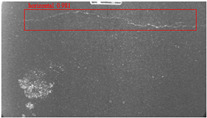	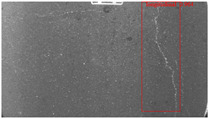	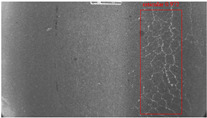
	Confidence: 0.981	Confidence: 0.964	Confidence: 0.972
Bicubic	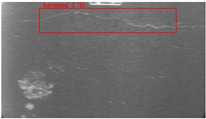	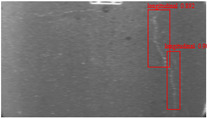	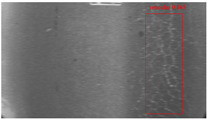
	Confidence: 0.783	Confidence: 0.892,0.905	Confidence: 0.865
SRCNN	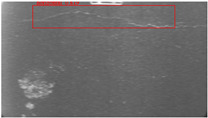	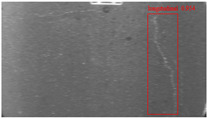	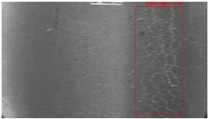
	Confidence: 0.819	Confidence: 0.954	Confidence: 0.916
DRCN	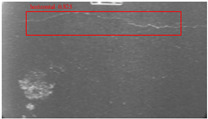	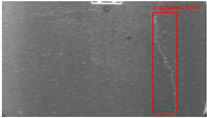	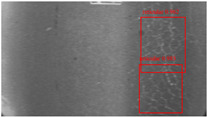
	Confidence: 0.825	Confidence: 0.934	Confidence: 0.962,0.985
SRGAN	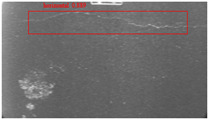	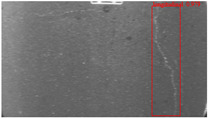	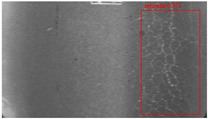
	Confidence: 0.889	Confidence: 0.979	Confidence: 0.971
Improved Method	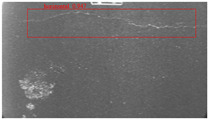	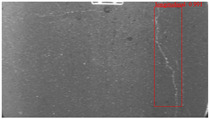	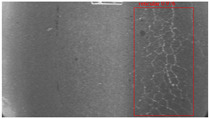
	Confidence: 0.947	Confidence: 0.991	Confidence: 0.976

**Table 7:** Comparison of crack segmentation F1 value and SSIM value of different methods.

Method	F1	SSIM
Bicubic	0.703	0.9823
SRCNN	0.711	0.9835
DRCN	0.723	0.9840
SRGAN	0.725	0.9902
**Improved method**	**0.737**	**0.9945**

## Data Availability

Some or all data, models, or code that support the findings of this study are available from the corresponding author upon reasonable request. The images from all sample sets used in this paper can be obtained from https://github.com/juhuyan/CrackDataset_DL_HY accessed on 10 October 2022 and http://www.cs.toronto.edu/~kriz/cifar.html. The modes and code used in this paper can be obtained from the corresponding authors after publication.
